# Role of oxytocin in modulating addictive behaviour

**DOI:** 10.1192/j.eurpsy.2021.99

**Published:** 2021-08-13

**Authors:** P. Bach, A. Koopmann, F. Kiefer

**Affiliations:** Department Of Addictive Behaviour & Addiction Medicine, Central Insitute of Mental Health, Mannheim, Germany

**Keywords:** alcohol use disorder, Funtional Magnetic Resonance Imaging, Psychopharmacology, oxytocin

## Abstract

**Background:**

The brain oxytocin system is involved in a wide range of addictive behaviors, inhibiting prime- and cue-induced relapse in preclinical models of substance use disorders. Animal studies linked oxytocin’s effects on drug ingestion to modulation of neurotransmission in the nucleus accumbens (NAc). We set out to investigate whether oxytocin can modulate alcohol cue-induced functional connectivity between the brain reward system and cortical regions.

**Methods:**

Fifteen male heavy social drinkers were enrolled in a randomized double-blind placebo-controlled cross-over functional magnetic resonance imaging study (fMRI) investigating the effect of 24 IU oxytocin on alcohol cue-modulated functional connectivity.

**Results:**

Results of the functional connectivity analyses show that oxytocin application significantly reduced connectivity between the NAc and cuneus and thalamo-occipital connectivity, while enhancing connectivity between the paracingulate gyrus and precentral gyrus (tow-sided seed-level false discovery rate p_FDR_ < 0.05). These effects were specific to the alcohol presentation and were absent during processing of neutral pictures. In addition, the NAc-cuneus connectivity significantly correlated with subjective alcohol cue-induced craving during the scanning session (r = 0.538, p = 0.024). Conclusion: Results provide initial evidence for condition-specific and significant attenuation of NAc connectivity by oxytocin in a sample of heavy social drinkers that was related to lower subjective alcohol craving during the fMRI task. Oxytocin-induced attenuation of NAc connectivity was specific to processing alcohol stimuli and might reflect an attenuation of alcohol-cue saliency by oxytocin that could lead to a reduction of the sensitivity towards the appetitive aspects of alcohol cues.

**Disclosure:**

No significant relationships.

